# Efficacy and Feasibility of Programmed Death-1/Programmed Death Ligand-1 Blockade Therapy in Non-Small Cell Lung Cancer Patients With High Antinuclear Antibody Titers

**DOI:** 10.3389/fonc.2021.610952

**Published:** 2021-03-15

**Authors:** Atsuto Mouri, Kyoichi Kaira, Ou Yamaguchi, Kosuke Hashimoto, Yu Miura, Ayako Shiono, Shun Shinomiya, Tomoe Akagami, Hisao Imai, Kunihiko Kobayashi, Hiroshi Kagamu

**Affiliations:** Department of Respiratory Medicine, Comprehensive Cancer Center, International Medical Center, Saitama Medical University, Saitama, Japan

**Keywords:** antinuclear antibodies, immune-check point inhibitor, non-small cell lung cancer, immune-related adverse events, feasibility

## Abstract

**Background:**

Immune checkpoint inhibitor (ICI) therapy has been described to markedly improve patient survival. However, reports describing the antitumor therapeutic efficacy and safety of ICIs in patients with autoantibodies are scarce.

**Methods:**

This study examined the efficacy and feasibility of ICIs in antinuclear antibody (ANA)-positive patients with non-small cell lung cancer (NSCLC). An ANA titer greater than 1:40 and 1:80 was defined as positive and high, respectively. Patients who were treated with ICIs at Saitama Medical University, International Medical Center between January 2016 and December 2018 were retrospectively reviewed.

**Results:**

One hundred and nineteen of the 266 patients (44.7%) who received nivolumab, pembrolizumab, and atezolizumab had positive ANA titers. Their median age was 69 (range, 39–84) years. The overall response rate of the ANA-positive patients was 35.9% (37/103), which was not less than that of the ANA-negative group. The median progression-free survival in the ANA-positive group was 6.3 months versus 4.3 months in the ANA-negative group (*p* = 0.08). Twenty-seven ANA-positive patients (10.2%) had high ANA titers. However, ICI efficacy was not decreased in these patients. Regardless of the cutoff of ANA titers (1:40 or 1:80), the rate of patients who experienced adverse events were not significantly different between the two groups.

**Conclusion:**

The administration of ICIs to ANA-positive patients has clinical benefits. The prevalence of adverse events in the ANA-positive group was not higher than that in the ANA-negative group.

## Introduction

Immune checkpoint inhibitors (ICIs) improve the survival of patients with advanced lung cancer (1–4). ICIs such as anti-programmed death-1 (PD-1)/programmed death ligand-1 (PD-L1) antibodies and cytotoxic T-lymphocyte-associated protein 4 (CTLA-4) are widely administered to patients with different kinds of neoplasms such as lung cancer, melanoma, renal cancer, urothelial cancer, and Hodgkin’s lymphoma (5–7). However, the management of adverse events is a critical issue, and this needs to be done carefully for the maximum efficacy of ICIs to be obtained. To avoid severe ICI toxicity, physicians should accurately judge the patient’s eligibility for ICI therapy. To prevent unnecessary immune-related adverse events (irAEs), the patient’s autoantibodies are often examined prior to treatment. Antinuclear antibody (ANA) is a key biomarker for evaluating autoimmune disorders (AIDs). Pre-existing AIDs can exacerbate irAEs. Thus, patients with AIDs are excluded from clinical trials. Khan et al. reported that 13.5% of 210,509 patients with lung cancer had AIDs, and majority of the patients were females, elderly, and had early stage disease (8). According to their study, rheumatoid arthritis (5.9%), psoriasis (2.8%), and polymyalgia rheumatica (1.8%) were the most common AIDs (8). It is common to find lung cancer patients with AIDs.

Checkpoint pathways such as PD-1, PD-L1, and CTLA-4 are mechanisms that regulate the immune response. Therefore, it is possible to encounter patients with AIDs during the administration of ICIs, requiring systemic steroid therapy. If there are any patients with active AIDs, ICIs should be avoided to prevent severe irAEs. Regarding patients with increased ANA titers and without active AIDs, it is still unclear whether the initiation of ICIs is clinically possible. Recently, several researchers have reported that it may be possible to administer ICIs without exacerbation of irAEs in patients with advanced non-small cell lung cancer (NSCLC) (9–12). However, the potential of ANA titer as a predictive marker for the efficacy and toxicity of ICIs is still controversial (9–12). As previous research has several limitations, such as limited sample sizes and varying ANA titer cutoffs, we cannot administer ICIs in NSCLC patients without AIDs based on increased ANA titers.

Based on this background, we retrospectively investigated the efficacy and tolerability of anti-PD-1 antibody monotherapy in advanced ANA-positive NSCLC patients without any AIDs.

## Materials and Methods

### Study Design and Patients

**Figure 1:**
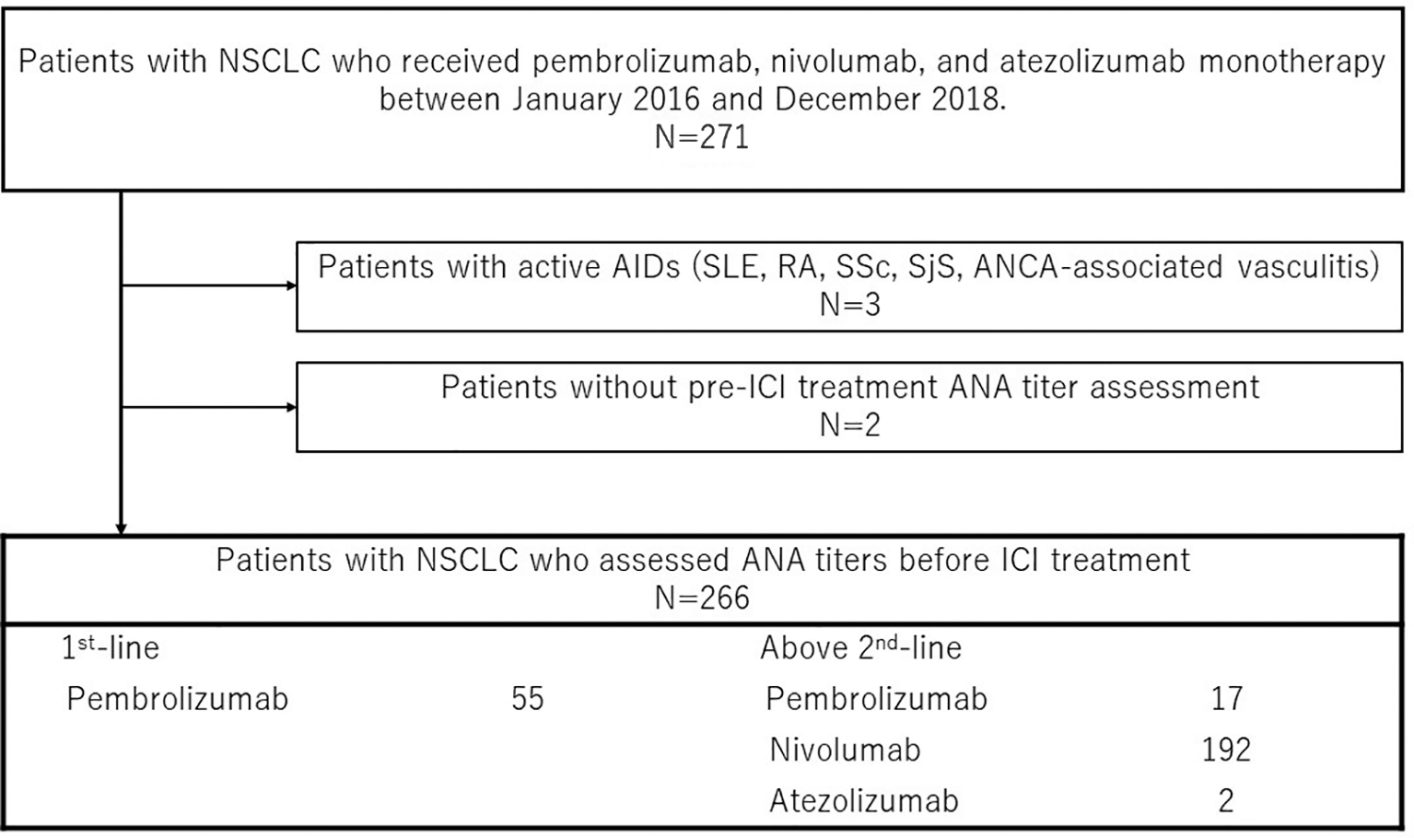
Study schema, antinuclear antibody (ANA) titers in 271 patients with advanced NSCLC were assessed before anti-PD-1/PD-L1 antibodies monotherapy administration at Saitama Medical University, International Medicine Center. Patients treated previously with other ICIs (Pembrolizumab and nivolumab including 1^st^-line therapy) or who did not have pre-treatment ANA titers before ICI or who had active major AIDs were excluded. Nivolumab, pembrolizumab, and atezolizumab were administered to 192, 72 (55 as 1^st^ setting), and 2 patients. PFS, progression-free survival; OS, overall survival; ICI, immune checkpoint inhibitors; PD-1, programmed death-1; PD-L1, programmed death ligand 1; ANA, antinuclear antibody.

Between February 2016 and December 2018, the data of 271 patients with advanced NSCLC who received anti-PD-1/PD-L1 antibody monotherapy (single-agent nivolumab, pembrolizumab, and atezolizumab) at Saitama Medical University, International Medicine Center were obtained from the hospital’s medical records. Of these 271 patients, 2 patients whose pre-treatment ANA titers were not available were excluded. Three patients with active rheumatoid arthritis were excluded. Finally, 266 patients were eligible for the study. **Figure 1** shows the patient selection consort diagram.

All procedures performed were in accordance with the ethical standards of the Institutional Ethics Committee of Saitama Medical University, International Medical Center and with the 1964 Declaration of Helsinki and its later amendments or comparable ethical standards.

### Assessment of Antinuclear Antibody and Rheumatoid Factor

Serum ANA titers were assessed before ICI initiation in all the patients. The ANA titer was measured by immunofluorescence at our institution. The ANA cutoff titer was set at 1:40 or 1:80. Because rheumatoid factor (RF) had been measured in 264 of the 266 enrolled before ICI treatment, we analyzed the survival efficacy according to high-grade RF using a cutoff of 15 IU/ml as an additional examination. The cut-off value of ANA and RF was defined according to previous studies. It is controversial which cut-off values of ANA are suitable for the survival analysis, 1:40 or 1:80. Thus, we analyzed using both cut-off points.

### Treatment and Evaluation

Nivolumab, pembrolizumab, and atezolizumab were administered intravenously at 3 mg/kg or 240 mg/day every 2 weeks, 200 mg/day every 3 weeks, and 1,200 mg/day every 3 weeks, respectively. Complete blood cell count, differential count, routine chemistry measurements, physical examination, and toxicity levels were evaluated. Acute toxicity was graded according to the Common Terminology Criteria for Adverse Events version 5.0. Tumor response was evaluated according to the Response Evaluation Criteria in Solid Tumors criteria ver. 1.1 (13).

### Assessment of Clinical Data

A baseline tumor assessment and response evaluation was carried out before ICI administration based on computed tomography (CT), magnetic resonance imaging, or proton emission tomography-CT findings according to the local standard of practice.

The overall response rate (ORR), defined as the proportion of patients who achieved a complete response (CR) or partial response (PR); and the disease control rate (DCR), defined as the proportion of patients who achieved CR, PR, or stable disease (SD) were evaluated in this study. Progression-free survival (PFS) was defined as the time from the initiation of ICI therapy to confirmed disease progression or death by any cause. PFS was censored at the date of the last follow-up visit for progression-free patients. OS was defined as the time from the initiation of ICI therapy to death due to any cause (event) or last contact (censored). The minimum follow-up period to ascertain the PFS and OS was 5 days after ICI therapy administration. At each follow-up, a routine physical examination, and laboratory and imaging investigations were done to assess safety. We obtained clinical information from the medical records without any protocol restriction.

### Statistical Analysis

P values < 0.05 were considered statistically significant. Fisher’s exact test, chi-squared, and Mann-Whitney U tests were used to examine the association between two categorical variables. The Kaplan–Meier method was used to estimate survival as a function of time, and survival differences were analyzed by log-rank tests. Patients with ANA titers above 1:40 were considered ANA-positive, and those with ANA titers > 1:80 were considered as having high ANA titers. Statistical analyses were performed using JMP 10 software, SASS. Corresponding confidence intervals were calculated using the Cox proportional hazards model.

## Results

### Patients’ Characteristics

There were 95 males (79.8%) and 24 females (20.2%). The median patient age was 69 (range, 39–84) years. A total of 119 (44.7%) patients were ANA-positive, while 27 (10.2%) had high ANA titers. Ninety-six patients (80.7%) had an Eastern Cooperative Oncology Group (ECOG) performance status (PS) of 0 or 1. Seventeen patients (14.3%) were never smokers. Of all patients, nivolumab, pembrolizumab, and atezolizumab were administered to 192, 72 (55 as 1^st^ setting), and 2 patients, respectively (**Figure 1**). **Table 1** shows the patient characteristics according to the ANA cutoff titer. Squamous cell carcinoma was observed in 26 cases (21.8%), adenocarcinoma in 71 (59.7%), and other histology types in 22 patients (18.5%). Epidermal growth factor receptor mutations were observed in 13 patients (10.9%) and rearrangement of anaplastic lymphoma kinase-echinoderm microtubule-associated protein-like 4 in 1 patient (0.8%). Nineteen patients (16.0%) experienced recurrence after operation or radiation therapy, and 23 (19.3%) and 77 (64.7%) patients had stage III and IV tumors, respectively. The tumor proportion score using PD-L1 immunohistochemistry (clone 22C3) was not significantly different between the ANA-positive and ANA-negative groups. As shown in **Table 1**, no significant differences in age, sex, PS, smoking history, tumor type, staging, mutation, PD-L1 status, and treatment lines were observed according to the ANA titer (1:40 or 1:80).

**Table 1:** Patient characteristics according to ANA titer.

Variables	All patients n = 266 (%)	ANA<40 n = 147 (%)	ANA≧40 n = 119 (%)	*p*-value	ANA<80 n = 239 (%)	ANA≧80 n = 27 (%)	*p*-value
Age [Median]	69 (31–86)	69 (31–86)	69 (39–84)		69 (31–86)	69 (43–79)	
Gender Male/Female	204/62 (76.7/23.3)	109/38 (74.1/25.9)	95/24 (79.8/20.2)	0.31	183/56 (76.6/23.4)	21/6 (77.8/22.2)	0.99
PS 0–1/Above 2	217/49 (81.6/18.4)	121/26 (82.3/17.7)	96/23 (80.7/19.3)	0.75	194/45 (81.2/18.8)	23/4 (85.2/14.8)	0.80
Smoking history Former/Never	217/49 (81.6/18.4)	115/32 (78.2/21.8)	102/17 (85.7/14.3)	0.15	192/47 (80.3/19.7)	25/2 (92.6/7.4)	0.19
Histology Adeno/Squamous/Others	150/67/49 (56.4/25.2/18.4)	79/41/27 (53.7/27.9/18.4)	71/26/22 (59.7/21.8/18.5)	0.51	132/61/46 (55.2/25.5/19.2)	18/6/3 (66.7/22.2/11.1)	0.47
Disease stage III/IV/Recurrence	45/172/49 (16.9/64.7/18.4)	22/95/30 (15.0/64.6/20.4)	23/77/19 (19.3/64.7/16.0)	0.50	38/156/45 (15.9/65.3/18.8)	7/16/4 (25.9/59.3/14.8)	0.42
Radiotherapy Radical/Palliative/None	40/65/161 (15.0/24.4/60.5)	25/37/85 (17.0/25.2/57.8)	15/28/76 (12.6/23.5/63.9)	0.53	35/61/143 (14.6/25.5/59.8)	5/4/18 (18.5/14.8/66.7)	0.42
Driver mutation Wild type/EGFR or ALK	225/41 (84.6/15.4)	120/27 (81.6/18.4)	105/14 (88.2/11.8)	0.17	200/39 (83.7/16.3)	25/2 (92.6/7.4)	0.40
PD-L1 (TPS) <1%/1–49%/≥50%/Unknown	10/15/63/178 (3.8/5.6/23.7/66.9)	5/9/30/103 (3.4/6.1/20.4/70.1)	5/6/33/75 (4.2/5.0/27.7/63.0)	0.53	10/14/56/159 (4.2/5.9/23.4/66.5)	0/1/7/19 (0.0/3.7/25.9/70.4)	0.91
Treatment lines 1st/2nd/Above 3rd	55/138/73 (20.7/51.9/27.4)	25/83/39 (17.0/56.5/26.5)	30/55/34 (25.2/46.2/28.6)	0.17	50/124/65 (20.9/51.9/27.2)	5/14/8 (18.5/51.9/29.6)	0.94

PS, performance status; ANA, antinuclear antibody; RT, radiotherapy; EGFR, epidermal growth factor receptor; ALK, Anaplastic lymphoma kinase; TPS, Tumor Proportion Score; adeno, adenocarcinoma; squamous, squamous cell carcinoma; PD-L1, programmed death ligand-1; Recurrence, recurrence after surgical resection.

The proportion of RF-positive patients at RF cutoff values of 5 IU/ml and 15 IU/ml were observed in 28.7% (77/268) and 12.3% (33/268), respectively.

### Efficacy and Survival Analysis According to the Antinuclear Antibody Titer

PR was observed in 37 patients (35.9%), SD in 32 patients (26.9%), and PD in 34 patients (28.6%). The ORR and DCR were 35.9% and 67.0%, respectively. **Table 2** shows the response rate of PD-1/PD-L1 blockade according to the ANA titer. The patients with positive and high ANA titers yielded an ORR of 35.9% and 43.5%, respectively, without any significant difference.

**Table 2:** Response rate of the ICI treatment according to ANA titer.

****Response****	****All patients**** n = 266	****ANA< 40**** n = 147	****ANA≧40**** n = 119	*****p*-value****	****ANA< 80**** n = 239	****ANA≧80**** n = 27	*****p*-value****
**CR**	1	1	0		1	0	
**PR**	71	34	37		61	10	
**SD**	73	41	32		66	7	
**PD**	93	59	34		87	6	
**NE**	28	12	16		24	4	
**ORR**	30.2%	25.9%	35.9%	0.21	28.8%	43.5%	0.25
**DCR**	60.9%	56.2%	67.0%	0.32	59.5%	73.9%	0.42

ICI, immune checkpoint inhibitor; ANA, antinuclear antibody; CR, complete response; PR, partial response; SD, stable disease; PD, progressive disease; NE, not evaluable; ORR, overall response rate; DCR, disease control rate.

**Figure 2:**
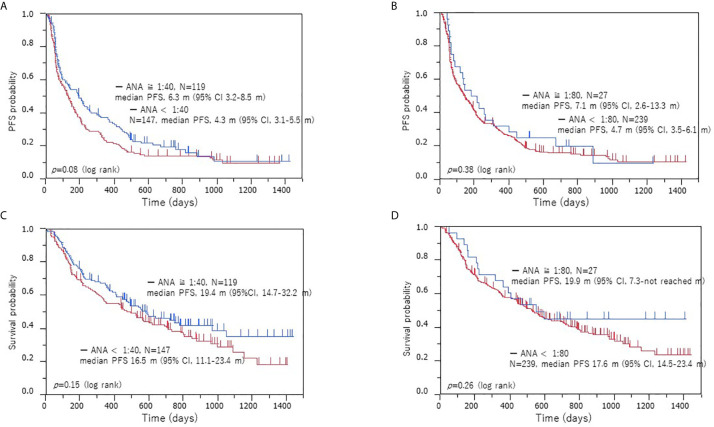
Kaplan–Meier survival curves of PFS and OS according to ANA positivity in the low and high cohorts. PFS **(A)** and OS **(B)** when a positive ANA cutoff of 1:40 was used, PFS **(C)** and OS **(D)** when a positive ANA cutoff of 1:80 was used. Survival analysis with ICI treatment: no significant difference in the PFS and OS was observed between the ANA-positive and ANA-negative patients or high and low ANA titer cohorts. PFS, progression-free survival; OS, overall survival; ANA, anti-nuclear antibody.

Next, we performed a survival analysis according to the ANA titer. Of 266 patients, 117 experienced recurrence after ICI initiation, and 76 died. **Figure 2** shows the Kaplan-Meier survival curve according to the different ANA cutoff values (1:40 and 1:80). The median PFS in the patients with positive and negative ANA were 6.3 and 4.3 months (*p* = 0.08), respectively, and the median OS was 19.4 and 16.5 months (*p* = 0.14), respectively (**Figures 2A, B**). The median PFS was 6.5 and 4.7 months (*p* = 0.42) in patients with high and low ANA titers, respectively, while the median OS was 18.3 and 17.6 months (*p* = 0.41), respectively (**Figures 2C, D**). In the survival analysis according to histology, PFS was longer in the ANA-positive patients with adenocarcinoma (*p* = 0.03) than in the ANA-negative patients, though no significant difference was observed in OS (*p* = 0.18) (**Supplementary Figure A1** **, online only**). No significant differences were observed in the PFS (*p* = 0.76) and OS (*p* = 0.63) between the ANA-positive and ANA-negative patients with non-adenocarcinoma tumors (**Supplementary Figure A1** **, online only**). However, no significant differences between the patients with high and low ANA titers in the PFS and OS were observed, regardless of the histological types (**Supplementary Figure A2** **, online only**).

Using an RF cutoff value of 15, the median PFS in RF-positive and RF-negative patients was 6.3 months and 4.7 months (*p* = 0.45), respectively, while the median OS was 11.5 months and 18.5 months (*p* = 0.66), respectively (**Supplementary Figure A3** **, online only**).

### Clinical Outcome Feasibility

**Figure 3:**
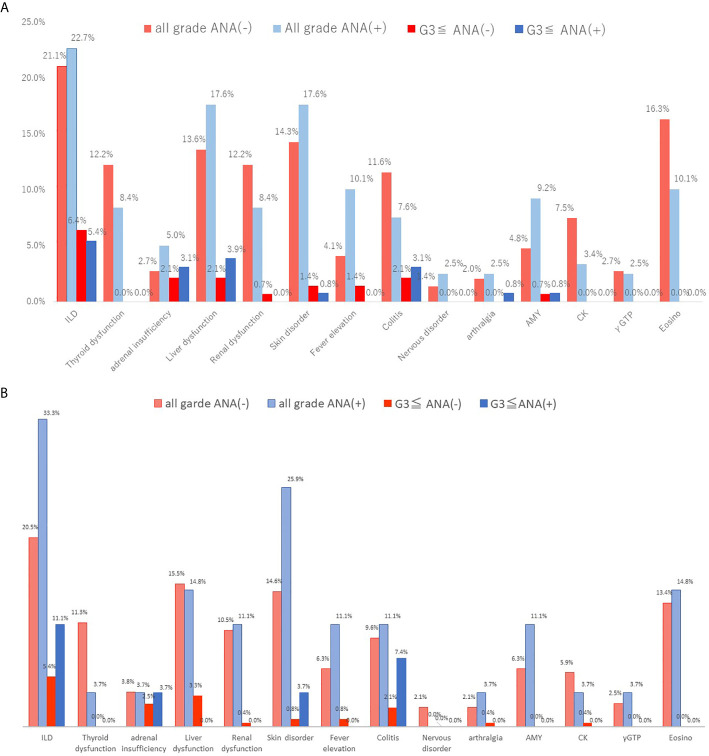
Immune-related adverse events between ANA-positive and ANA-negative patients (cutoff of 1:40) or high and low ANA (cutoff of 1:80). The irAEs were listed at frequency 2% or more in either group. **(A)** When a positive ANA cutoff of 1:40 was used, **(B)** when a positive ANA cutoff of 1:80 was used. irAEs, immune-related adverse events; AMY, amylase; CK, creatine kinase; γGTP, γ- Glutamyltranspeptidase.

**Table 3** shows the comparison of irAEs according to the ANA cutoff value. There were no significant differences in the frequency of irAEs between ANA-positive and ANA-negative patients (**Table 3** and **Figure 3A**) and between those with high and low ANA titers (**Table 3** and **Figure 3B**). The frequency of steroid therapy in irAEs was 26.0% (31/119) in ANA-positive patients and 24.8% (35/148) in ANA-negative patients (*p* = 0.78) (**Supplementary Table A8**, **online only**). Likewise, a similar trend was observed between the patients with high and low ANA titers (**Supplementary Table A8**, **online only**). One ANA-positive patient experienced Sjögren syndrome (SjS) related to ICI use.

**Table 3:** Immune-related adverse events.

	All grade (ANA 1:40)				>≧Grade3 (ANA 1:40)				>All grade (ANA 1:80)				>≧Grade3 (ANA 1:80)			
Variables	All patients	ANA<40	ANA≧40	* *	All patients	ANA<40	ANA≧40	* *	All patients	ANA<80	ANA≧80	* *	All patients	ANA<80	ANA≧80	* *
	n = 266 (%)	n = 147 (%)	n = 119 (%)	*p*-value	n = 266 (%)	n = 147 (%)	n = 119 (%)	*p*-value	n = 266 (%)	n = 239 (%)	n = 17 (%)	*p*-value	n = 266 (%)	n = 239 (%)	n = 17 (%)	*p*-value
**ILD**	58 (21.8)	31 (21.1)	27(22.7)	0.77	16 (6.0)	9 (6.1)	7 (5.9)	0.99	58 (21.8)	49 (20.5)	9 (33.3)	0.14	16 (6.0)	13 (5.4)	3 (11.1)	0.21
**Hypothyroidism**	28 (10.5)	18 (12.2)	10(8.4)	0.32	0 (0)	0 (0)	0 (0)	0.99	28 (10.5)	27 (11.3)	1 (3.7)	0.33	0 (0)	0 (0)	0 (0)	0.99
**Adrenal insufficiency**	10 (3.8)	4 (2.7)	6(5.0)	0.35	7 (2.6)	3 (2.0)	4 (3.4)	0.7	10 (3.8)	9 (3.8)	1 (3.7)	0.99	7 (2.6)	6 (2.5)	1 (3.7)	0.53
**Liver dysfunction**	41 (15.4)	20 (13.6)	21(17.6)	0.4	8 (3.0)	3 (2.0)	5 (4.2)	0.47	41 (15.4)	37 (15.5)	4 (14.8)	0.78	8 (3.0)	8 (3.3)	0 (0)	0.99
**Renal dysfunction**	28 (10.5)	18 (12.2)	10(8.4)	0.32	1 (0.4)	1 (0.7)	0 (0)	0.99	28 (10.5)	25 (10.5)	3(11.1)	0.92	1 (0.4)	1 (0.4)	0 (0)	0.99
**Skin disorder**	42 (15.8)	21 (14.3)	21(17.6)	0.5	3 (1.1)	2 (1.4)	1 (0.8)	0.99	42 (15.8)	35 (14.6)	7 (25.9)	0.17	3 (1.1)	2 (0.8)	1 (3.7)	0.28
**Fever**	18 (6.8)	6 (4.1)	12(10.1)	0.08	2 (0.8)	2 (1.4)	0 (0)	0.5	18 (6.8)	15 (6.3)	3 (11.1)	0.41	2 (0.8)	2 (0.8)	0 (0)	0.99
**Colitis**	26 (9.8)	17 (11.6)	9(7.6)	0.31	7 (2.6)	3 (2.0)	4 (3.4)	0.7	26 (9.8)	23 (9.6)	3 (11.1)	0.74	7 (2.6)	5 (2.1)	2 (7.4)	0.15
**Nervous system disorder**	5 (1.9)	2 (1.4)	3(2.5)	0.66	0 (0)	0 (0)	0 (0)	0.99	5 (1.9)	5 (2.1)	0 (0)	0.99	0 (0)	0 (0)	0 (0)	0.99
**Arthralgia**	6 (2.3)	3 (2.0)	3(2.5)	0.99	1 (0.4)	0 (0)	1 (0.8)	0.45	6 (2.3)	5 (2.1)	1 (3.7)	0.48	1 (0.4)	1 (0.4)	0 (0)	0.99
**AMY**	18 (6.8)	7 (4.8)	11(9.2)	0.22	0 (0)	0 (0)	0 (0)	0.99	18 (6.8)	15 (6.3)	3 (11.1)	0.41	0 (0)	0 (0)	0 (0)	0.99
**CK**	15 (5.6)	11 (7.5)	4(3.4)	0.19	1 (0.4)	1 (0.7)	0 (0)	0.99	15 (5.6)	14 (5.9)	1 (3.7)	0.99	1 (0.4)	1 (0.4)	0 (0)	0.99
**γ-GTP**	7 (2.6)	4 (2.7)	3(2.5)	0.99	0 (0)	0 (0)	0 (0)	0.99	7 (2.6)	6 (2.5)	1 (3.7)	0.53	0 (0)	0 (0)	0 (0)	0.99
**Eosinophil**	36 (13.5)	24 (16.3)	12(10.1)	0.15	0(0)	0 (0)	0 (0)	0.99	36 (13.5)	32 (13.4)	4 (14.8)	0.77	0 (0)	0 (0)	0 (0)	0.99

The irAE was listed at frequency 2% or more in either group.

ANA, antinuclear antibody; ILD, interstitial lung injury; AMY, amylase; CK, creatine kinase; γGTP, γ- Glutamyl transpeptidase.

## Discussion

This study is the largest study to verify the efficacy and toxicity of anti-PD-1/PD-L1 therapies in ANA-positive NSCLC patients. We found that the effectiveness and prevalence of irAEs were consistent regardless of the ANA titer. Even with high ANA titers, the usefulness of ICI was unrelated to the occurrence of AIDs, and the presence of ANA did not affect the efficacy and outcome after the PD-1/PD-L1 blockade treatment. According to our results, the proportion of ANA-positive NSCLC patients was similar to that in the healthy population (14). In this study, the frequency of ANA positivity was 44.7% (119/266) with the cutoff value at 1:40 and 10.2% (27/266) with the cutoff value at 80. Previous research shows that the presumptive rate of ANA positivity in a healthy population is 31.7% in individuals at 1:40 serum dilution, 13.3% at 1:80, 5.0% at 1:160, and 3.3% at 1:320 (14). Our study results indicated that the ANA positivity rate in patients with NSCLC was similar to that in a healthy population. Thus, serum ANA levels may not correlate with an increased risk of cancer (15).

In our study, the positive rates of RF at cutoff values of 5 IU/ml and 15 IU/ml before ICI treatment were recognized in 28.7% (77/268) and 12.3% (33/268), respectively. It has been already reported that RF yielded positive findings of 5%–25% in patients with malignant disease (16). Compared with previous investigations, the positive rates of ANA and RF in patients with NSCLC were similar to that of the healthy population. Aside from ANA, we investigated the efficacy according to the level of RF. However, there was no relationship between the expression of RF and ICI therapeutic efficacy in lung cancer.

Recently, five reports including our study described ICI efficacy and toxicity, and patient survival according to the ANA titer (9–12). Yoneshima et al. reported that the incidence of irAEs did not differ between patients with positive and negative ANA, and PFS and OS were significantly shorter in patients with positive ANA than in those with negative ANA (9). In their study, the ANA cutoff titer was defined as 1:40, and 21.7% (18/83) of all patients with advanced NSCLC were positive for ANA (9). They discussed why there was no difference in the ORR of PD-1 blockade between the positive and negative ANA groups, despite the worse survival of the positive ANA group. The frequency of the ANA-positive patients with a cutoff value of 1:40 in our study was 45%, which substantially differed from 21.7% in their study. Our study indicated no significant difference in the ORR for PD-1 blockade according to the ANA cutoff titer of 1:40 or 1:80. Generally, the ORR of PD-1 inhibitor is considered to reflect survival. Therefore, a small sample size of 83 patients in their study may have biased their results. Sakakida et al. investigated 191 patients treated with PD-1 blockade monotherapy, and the patients were divided into positive and negative ANA groups according to the cutoff value of 1:160 (10). Although a positive ANA titer was observed in 4.7% (9/191) in their study, the incidence of irAEs except for colitis, the ORR and survival did not differ between the groups (10). Toi et al. assessed the efficacy and safety of PD-1 blockade in 137 patients with advanced NSCLC based on the existence of preexisting autoimmune markers such as rheumatoid factor, ANA, antithyroglobulin, and anti-thyroid peroxidase (11). The results of their study indicated that PFS was significantly longer in patients with RF than in those without, but not significantly different between ANA-positive and ANA-negative patients. The ANA cutoff titer in their study was 1:40. Moreover, the development of irAEs and the ORR did not differ between the patients with positive and negative ANA titers (11). Giannicola et al. reported that patients with ANA, antibodies to extractable nuclear antigens, and anti-smooth muscle antibodies exhibited significantly better PFS and OS than those without those antibodies (12). However, it remains unclear whether ANA alone could predict the outcome and the exacerbation of irAEs after PD-1/PD-L1 blockade. Meanwhile, we investigated the efficacy and toxicity of ICI treatment according to histological type (adenocarcinoma or non-adenocarcinoma). Although the PFS of patients receiving ICI was not different between the high and low groups at the ANA cutoff titer of 1:80, it was significantly better in the positive group than in the negative group when using a cutoff of 1:40. This finding is in contrast to the results of previous studies wherein the ICI efficacy at an ANA cutoff titer of 1:40 was controversial. Although little is known about ICI treatment between positive and negative ANA titers according to histological type, the ANA cutoff titer of 1:40 may not be suitable for the analysis based on the ANA titer. Considering the results of these previous studies and ours, we can consider that the presence of ANA was not a significant predictor of the efficacy, patient survival, and irAEs after PD-1/PD-L1 blockade monotherapy. In the absence of AIDs, the administration of PD-1/PD-L1 blockade is feasible regardless of the ANA titers.

However, autoantibodies are not necessarily specific to the diagnosis of AIDs, and some additional laboratory investigations are needed for its diagnosis. Soyoung et al. discussed the intervention of regulatory T cells characterized by the expression of CD4+, CD25+, and Foxp3+ in patients with rheumatoid arthritis (17). It has been reported that the number or function of regulatory T cells decreases according to the severity of active SLE (18, 19). Leonardi et al. carried out a retrospective study in which 23% of 56 NSCLC patients with a history of AIDs experienced AID exacerbation after ICI treatment (20). A recent review described that the frequency of adverse events related to ICI therapy was not different between patients with and without AIDs (21). In the patients with AIDs who received ICI, AID exacerbation was observed in a few patients, and the incidence of irAEs and ICI efficacy was almost similar to those of previous clinical trials.

Our study has the largest sample size compared to previous studies. However, there are several limitations to our study. First, our study was a retrospective investigation; thus, it may have biased the results of our study. Second, we could not obtain adequate information on PD-L1 expression; therefore, little is known about the relationship between PD-L1 and ANA. Toi et al. described no close correlation between PD-L1 and ANA (11). Finally, our study did not include patients with AIDs who received ICI treatment. In our institute, we experienced 3 cases of AIDs who were treated with ICI (data not shown). All three patients contracted rheumatoid arthritis before ICI treatment, and one patient had SjS in addition to pre-existing rheumatoid arthritis without requiring administration of systemic steroids after obtaining polymyalgia rheumatica by ICI treatment. The other patients with rheumatoid arthritis did not experience exacerbation of their connective tissue disease. None of the patients who had existing AIDs before ICI treatment experienced serious irAEs (≥ grade 3). According to previous studies, ICIs may be used with adequate caution in patients with AIDs. Further studies should focus on the efficacy and safety of ICI treatment in patients with AIDs.

In conclusion, the efficacy of ICIs and incidence of irAEs after ICI initiation were not absolutely affected by high ANA titers. ICIs can be administered even in patients with high ANA titers and without AIDs. The potential of ANA as a predictive marker for NSCLC patients treated with ICI remains unclear. Physicians should consider the therapeutic possibility of ICI regardless of the ANA titer.

## Data Availability Statement

The original contributions presented in the study are included in the article/**Supplementary Material**. Further inquiries can be directed to the corresponding author.

## Ethics Statement

All procedures performed were in accordance with the ethical standards of the Institutional Ethics Committee of Saitama Medical University, International Medical Center and with the 1964 Declaration of Helsinki and its later amendments or comparable ethical standards.

## Author Contributions

AM, KyK, and OY conceptualized the study and prepared the manuscript. KH, YM, AS, SS, TA, and HI were in charge of patient management. OY and KuK performed the statistical analysis and collected the patient data. AM, OY, KyK, KH, and HK revised the manuscript. All authors contributed to the article and approved the submitted version.

## Conflict of Interest

AM, KyK, OY, and HK received research grants and received honorarium as speakers from the Ono Pharmaceutical Company, Bristol-Myers Company, and Chugai Pharmaceutical Company.

The remaining authors declare that the research was conducted in the absence of any commercial or financial relationships that could be construed as a potential conflict of interest.

## Supplementary Material

Supplementary Figure 1 Kaplan-Meier survival curves of PFS and OS in the positive and negative ANA cohorts when a positive ANA cutoff of 1:40 was used. **(A)** PFS in patients with non-adenocarcinoma tumors, **(B)** OS in patients with non-adenocarcinoma tumors, **(C)** PFS in patients with adenocarcinoma, **(D)** OS in patients with adenocarcinoma PFS, progression-free survival; OS, overall survival; ANA, anti-nuclear antibody.

Supplementary Figure 2 Kaplan-Meier survival curves of PFS and OS in the positive and negative ANA cohorts with a positive ANA cutoff of 1:80. **(A)** PFS in patients with non-adenocarcinoma tumors, **(B)** OS in patients with non-adenocarcinoma tumors, **(C)** PFS in patients with adenocarcinoma, **(D)** OS in patients with adenocarcinoma PFS, progression-free survival; OS, overall survival; ANA, anti-nuclear antibody.

Supplementary Figure 3 Kaplan-Meier survival curves of PFS and OS in the positive and negative RF cohorts when a positive RF cutoff of 15 was used. Survival analysis with ICI treatment: no significant difference in the PFS and OS was observed between the RF-positive and RF-negative patients. PFS, progression-free survival; OS, overall survival; RF, rheumatoid factor.

Supplementary Figure 4 Kaplan-Meier survival curves of PFS and OS in both positive ANA (≧1:40) and RF (≧15) or not. Survival analysis with ICI treatment: no significant difference in the PFS and OS was observed between both ANA and RF-positive and negative patients. PFS, progression-free survival; OS, overall survival; ANA, anti-nuclear antibody; RF, rheumatoid factor.
