# Supplementary figures and images for: Efficacy and Feasibility of Programmed Death-1/Programmed Death Ligand-1 Blockade Therapy in Non-Small Cell Lung Cancer Patients With High Antinuclear Antibody Titers
Source: Front Oncol. 2021 Mar 15;11:610952. doi: 10.3389/fonc.2021.610952 (PMC8005657; doi:10.3389/fonc.2021.610952)

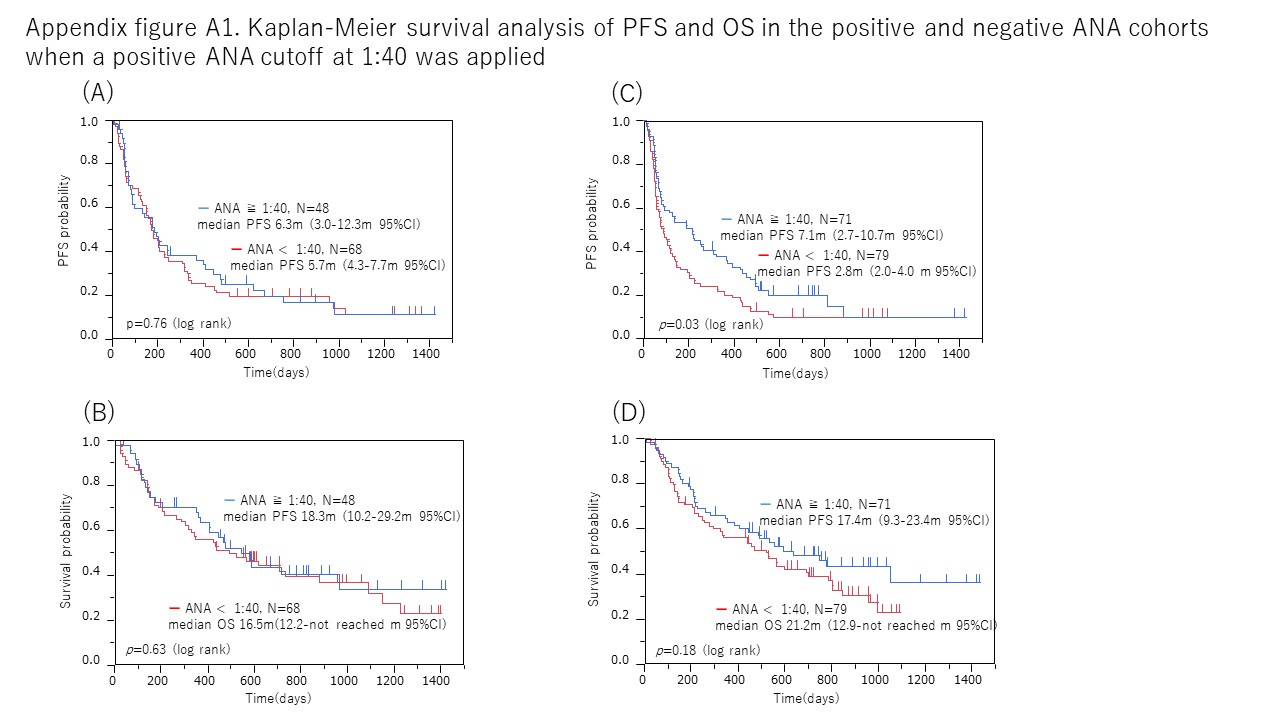

Supplement: Supplementary Figure 1 — Kaplan-Meier survival curves of PFS and OS in the positive and negative ANA cohorts when a positive ANA cutoff of 1:40 was used. (A) PFS in patients with non-adenocarcinoma tumors, (B) OS in patients with non-adenocarcinoma tumors, (C) PFS in patients with adenocarcinoma, (D) OS in patients with adenocarcinoma PFS, progression-free survival; OS, overall survival; ANA, anti-nuclear antibody. [file Image_1.jpeg]

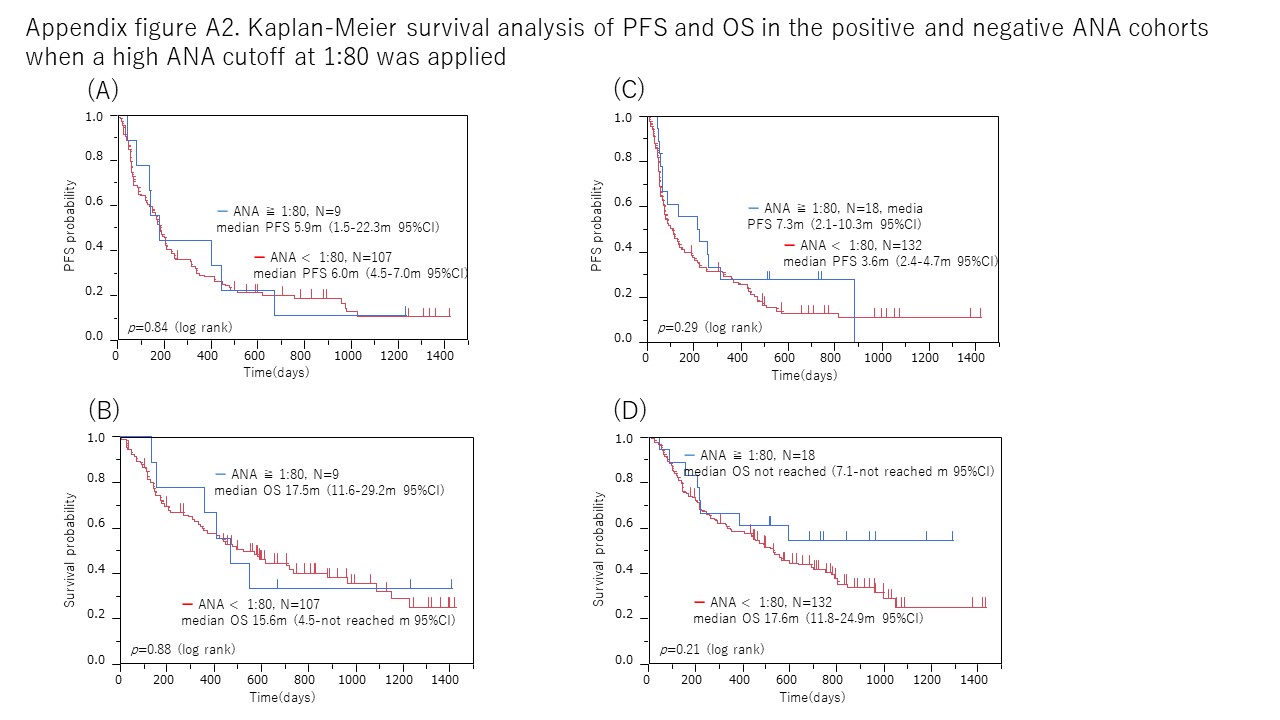

Supplement: Supplementary Figure 2 — Kaplan-Meier survival curves of PFS and OS in the positive and negative ANA cohorts with a positive ANA cutoff of 1:80. (A) PFS in patients with non-adenocarcinoma tumors, (B) OS in patients with non-adenocarcinoma tumors, (C) PFS in patients with adenocarcinoma, (D) OS in patients with adenocarcinoma PFS, progression-free survival; OS, overall survival; ANA, anti-nuclear antibody. [file Image_2.jpeg]

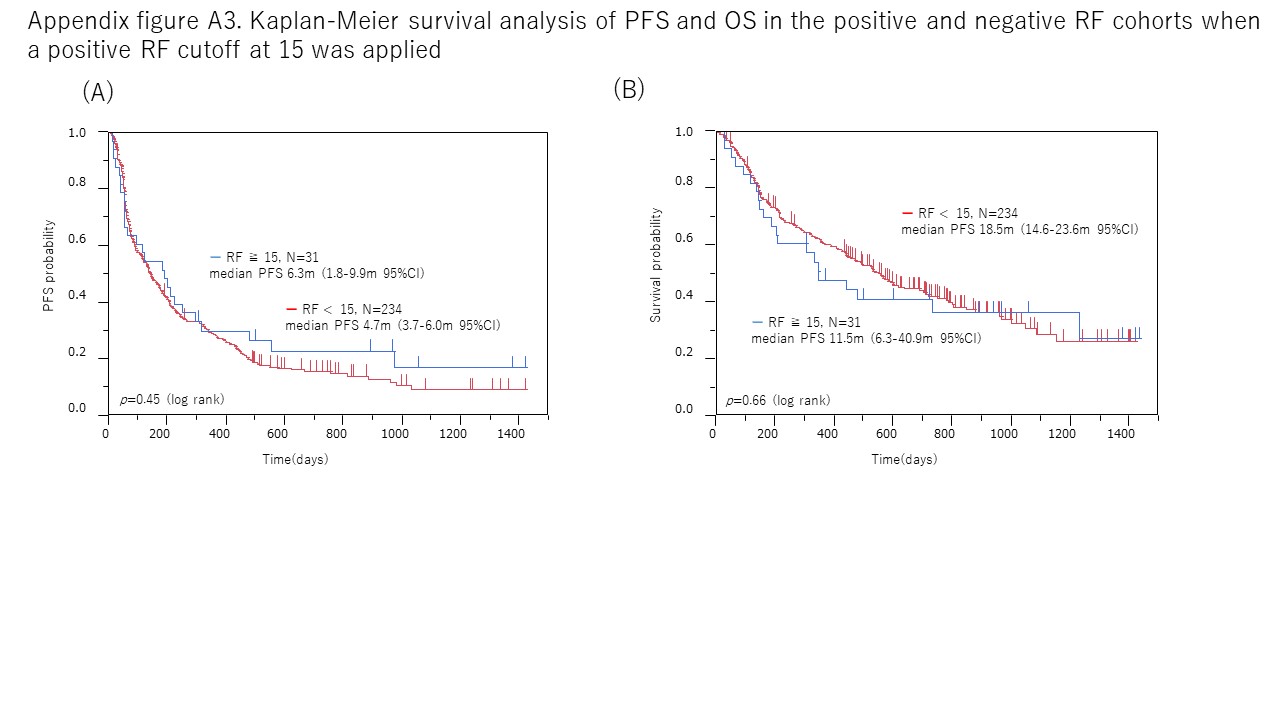

Supplement: Supplementary Figure 3 — Kaplan-Meier survival curves of PFS and OS in the positive and negative RF cohorts when a positive RF cutoff of 15 was used. Survival analysis with ICI treatment: no significant difference in the PFS and OS was observed between the RF-positive and RF-negative patients. PFS, progression-free survival; OS, overall survival; RF, rheumatoid factor. [file Image_3.jpeg]

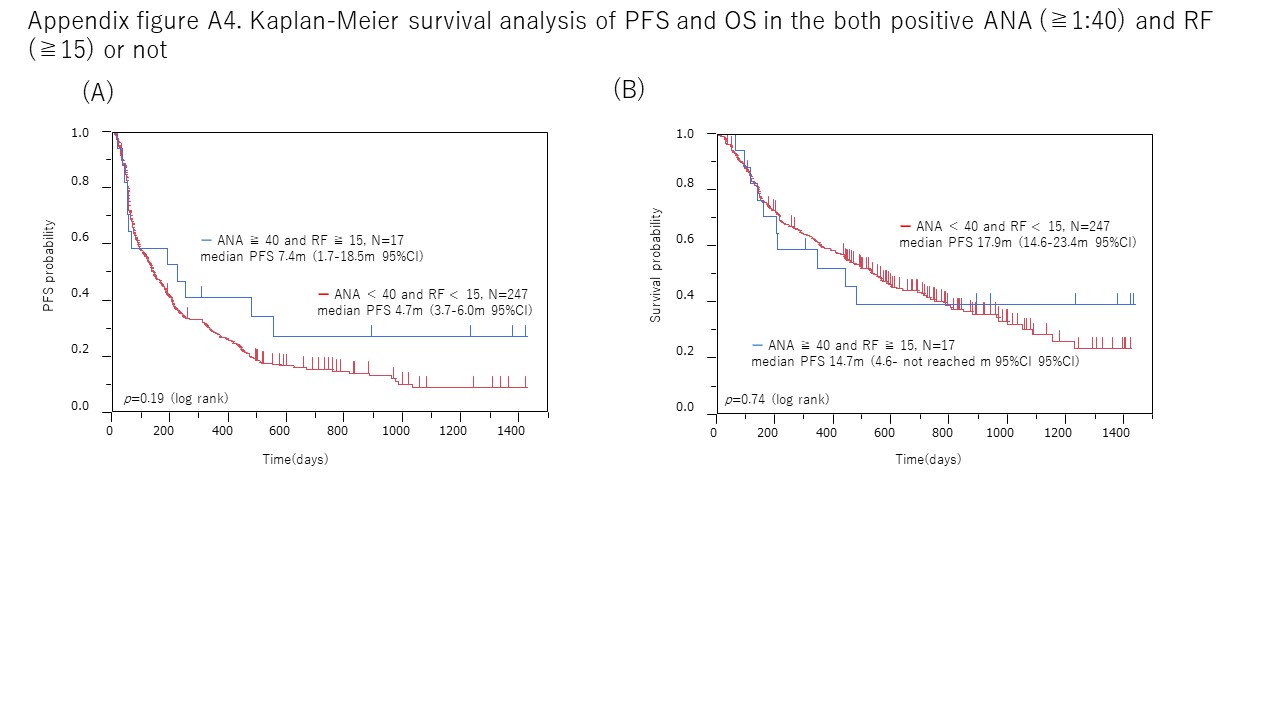

Supplement: Supplementary Figure 4 — Kaplan-Meier survival curves of PFS and OS in both positive ANA (≧1:40) and RF (≧15) or not. Survival analysis with ICI treatment: no significant difference in the PFS and OS was observed between both ANA and RF-positive and negative patients. PFS, progression-free survival; OS, overall survival; ANA, anti-nuclear antibody; RF, rheumatoid factor. [file Image_4.jpeg]
